# Implementation of Plum Skin as a Structuring Agent in Plum Spread

**DOI:** 10.3390/foods14040697

**Published:** 2025-02-18

**Authors:** Aleksandra Bajić, Biljana Cvetković, Jasna Mastilović, Miroslav Hadnađev, Marijana Djordjević, Miljana Djordjević, Bojana Filipčev

**Affiliations:** 1 Institute of Food Technology, University of Novi Sad, Bulevar Cara Lazara 1, 21000 Novi Sad, Serbia; biljana.cvetkovic@fins.uns.ac.rs (B.C.); miroslav.hadnadjev@fins.uns.ac.rs (M.H.); marijana.djordjevic@fins.uns.ac.rs (M.D.); miljana.djordjevic@fins.uns.ac.rs (M.D.); bojana.filipcev@fins.uns.ac.rs (B.F.); 2 BioSense Institute, University of Novi Sad, Dr Zorana Đinđića 1, 21000 Novi Sad, Serbia; jasna.mastilovic@biosense.rs

**Keywords:** fibers, pectin, rheology, texture, plum by-product, functional food

## Abstract

Plum skin, a by-product of industrial plum juice production, is rich in phenolic bioactives, functional compounds, and dietary fibers. These compounds support health, while the fibers may also act as structuring agents in food processing. This study investigated the structuring properties of lyophilized plum skin (LPS) in functional plum spreads produced in laboratory (F-LS) and semi-industrial (F-IS) environments, compared to a control spread (CS). Textural and rheological properties were analyzed through penetration, spreadability, flow, and dynamic oscillatory tests. Total, soluble, and insoluble dietary fibers (TDF, SDF, and IDF) in LPS and plum purée (PP) were measured using the enzymatic gravimetric method, and pectic substances contents were quantified using the colorimetric carbazole method. Fourier transform infrared spectroscopy confirmed the presence of polysaccharides and pectins in LPS. LPS had higher TDF, SDF, and IDF compared to PP, with TDF in LPS at 38.98 ± 0.52 g/100 g d.m. and IDF as the predominant fraction. The pectin content in LPS was 0.73 ± 0.03 g/100 g d.m., and water retention capacity ranged from 3.63 to 3.86 g/g depending on temperature (room, 50, and 82 °C). Incorporating LPS into the F-IS spread significantly increased all textural and rheological parameters, with TDF three times higher (6.69 g/100 g) compared to CS. All samples exhibited viscoelastic gel-like behavior, and LPS was a statistically significant structuring agent in both functional spreads compared to CS.

## 1. Introduction

With increasing awareness of the impact food choices have on health, the development of functional foods has captured significant attention from both consumers and the food industry. Despite the lack of a single, universally accepted definition of functional food, Temple proposed the following new definition [1]: “Functional foods are novel foods that have been formulated so that they contain substances or live microorganisms that have a possible health-enhancing or disease-preventing value, and at a concentration that is both safe and sufficiently high to achieve the intended benefit. The added ingredients may include nutrients, dietary fiber, phytochemicals, other substances, or probiotics”. Popular functional food items on the market include yogurt, cereals, margarine and butter, in addition to energy and protein bars and drinks [2]. Alongside functional foods, there has been increasing interest in foods designed for specific dietary purposes, such as light or low-fat options [2].

This trend also applies to the category of fruit-based gelled products, such as jams, jellies, marmalades, and “*pekmez*” (similar to fruit butter). According to EU regulation (Directive 2001/113/EC, 2001), those products must contain at least 60% sugar to qualify for specific labeling [3]. Reduced-sugar or low-calorie fruit jams, jellies, and marmalades, which may contain alternative sweeteners or less sugar than standard products, are permitted under the directive as long as they meet the required minimum fruit content (e.g., at least 35% fruit for jam). Global consumer demands for functional and minimally processed food have led to the production of reduced/low-calorie healthier alternatives to traditional products, such as fruit spreads. The gelling process of those fruit preparations involves the addition of pectin and other hydrocolloids to achieve a gel network in the system. The addition of gelling agents is essential to ensure the appropriate consistency of jams and similar products and that the fruit-pulp-sugar system remains stable within the product’s macrostructure [4].

The rising awareness of the health benefits of fiber-rich foods has driven the demand for new sources of dietary fiber. Recent research has shown that a fiber-rich diet can positively impact digestive health, cardiovascular health, weight control, and blood sugar regulation while also playing a key role in preventing chronic diseases, maintaining a healthy gut microbiome (acting as a prebiotic), ultimately contributing to overall well-being [5].

Aligning with the circular economy concept, by-products represent a new reservoir of functional materials and ingredients. By-products from fruits, vegetables, cereals, or algae processing are significant fiber sources and can be used in value-added products. Processing fruits and vegetables can result in 10–35% biowaste, typically in the form of pomace, which is rich in total dietary fibers and its soluble fraction [6,7]. The fiber content of pomace obtained after juice processing can range from 40% to 70%, depending on the type of fruit and extraction procedure [8]. Specifically, plum skin, a by-product of juice production, is recognized as a source of fiber, with concentrations reaching up to 49% in dried skin [8,9].

The physicochemical properties of fibers (e.g., water-retention capacity—WRC, viscosity, gel-forming ability, and fat-binding capacity) play a fundamental role in their further application in food processing [10]. Fibers can modify textural properties, reduce the risk of syneresis (liquid separation), act as stabilizers and emulsifiers, serve as thickening and gelling agents, replace fats in low and reduced-calorie food, and contribute to extending the shelf life of food products [5]. Pectin is categorized as soluble fiber and recognized as a rheological modifier [11] and used as a stabilizer, emulsifier, thickener, film-forming, gelling and glazing agent in the food industry [12,13].

Fruit jams, jellies, and similar fruit products are a suitable matrix for integrating fibers into the food structure [14]. Fibers added to new functional foods such as fruit jellies can modify viscoelastic and mechanical properties, color, and sensory properties [14]. Including novel ingredients in commercial foods is challenging since it can result in a less attractive texture for consumers [15]. Fibers from various sources (apples, bamboo, psyllium and wheat) can be added as functional ingredients in fruit jellies [14]. Pectins from passion fruit peel can serve as probiotic carriers in beverages that are beneficial for lactose-intolerant consumers [16]. It has been highlighted that the water-binding capacity of the plum skin is sufficient for its use as a food ingredient despite the soluble fraction loss during an enzymatic step in juice processing [8]. The concept of using lyophilized plum skin as a functional ingredient and hydrocolloid source needed for gelation was confirmed in our previous work [17]. Thus, an optimal reduced-calorie product was prepared without adding amidated low-methoxyl pectin (E440).

Given the factors outlined above, the objective of this study is to gain a deeper understanding of the macro- and microstructural, rheological, and textural properties of plum spreads produced in laboratory and semi-industrial environments. Since dietary fibers, particularly soluble fractions such as pectin, are known to act as texturizing agents, the plum materials were also characterized in terms of total dietary fiber (TDF), soluble dietary fiber (SDF), insoluble dietary fiber (IDF), and pectic fractions. In addition, FTIR analysis of lyophilized plum skin (LPS) obtained from the Serbian domestic cultivar “Čačanska rodna” was conducted. The hypothesis that plum-based spreads, enriched with dietary fiber from plum pomace, would have higher fiber content and lower calorie content compared to conventional commercial products was tested.

## 2. Materials and Methods

### 2.1. Plum Materials

The plum materials used in fruit product preparation were sourced from the “Čačanska rodna” plum cultivar. Blanched plum purée (PP) and lyophilized plum skin (LPS) were prepared as previously described with modifications to the drying procedure [17]. The 16-h drying process was conducted in the industrial lyophilizer (FD 100, PIGO-Belgrade, Serbia) under a vacuum of 99.8–99.9 kPa, with evaporator temperatures ranging from −45 °C to −50 °C. The obtained dried pomace (skin) was milled using a Foss Knifetec Sample Mill (FOSS, Hilleroed, Denmark) into LPS powder with a particle size ranging from 0.2 to 0.4 mm, while the dry matter content was 93.6%

### 2.2. Fruit Products Preparation

Plum spreads with LPS was manufactured, as explained previously [17,18]. The optimal functional spread (70% PP, 15% sucrose, 10% LPS, 5% water; *w*/*w*) was prepared in a laboratory (F-LS) environment in an open cooking pan at atmospheric pressure, while the industrial-scale functional spread (F-IS) was cooked in a semi-industrial vacuum cooker (Compconsult, Niš, Serbia). The control spread (CS) was prepared under vacuum, the same as F-IS, with the formulation modified by substituting the LPS with an equal amount of water (*w*/*w*). The formulations and processing conditions of all three spreads are presented in Table 1. Total soluble solids (TSS) were approximately 40 °Brix for both F-LS and F-IS, and 43 °Brix for CS, while the pH was 3.5 in all spreads [18]. F-IS and CS were filled in jars (370 mL) and pasteurized (85 °C; 1 h).

### 2.3. Determination of SDF, IDF and TDF

Contents of SDF, IDF and TDF were determined in raw materials LPS and PP, as well as in the final functional and control spread (F-IS and CS, respectively), according to AOAC 991.43 and AACC 32-07.01 [19,20].

### 2.4. Pectic Substances’ Determination

The pectic substances (pectin, pectic acids and protopectin) in the raw materials (LPS and PP) were determined using the colorimetric carbazol method [21], with modifications regarding the reduction of reagent and extract volumes.

### 2.5. Water Retention Capacity

The WRC of LPS powder was assessed using a method previously described [22]. LPS powder (2 g) was mixed with 30 mL of water at room temperature and left for 18 h. Afterward, the samples were heated to two different temperatures (50 °C and 82 °C), cooled to room temperature, centrifuged (20 min, 3000 *g*), and the mass of the precipitate was measured. Temperatures of 50 °C and 82 °C correspond to those in the manufacturing process (F-LS and F-IS, respectively).

### 2.6. ATR-Fourier Transform Infrared Spectroscopy (FTIR) Analysis of LPS

The Alpha II (Bruker Optics, Ettlingen, Germany) spectrometer equipped with a Platinum ATR Module consisting of single-reflectance diamond crystal was used for the acquisition of LPS spectra. Measurement was performed at room temperature within the scanning range 4000–400 cm^−1^, with a resolution of 4 cm^−1^, and spectra were obtained based on 24 scans.

### 2.7. Rheological Characterization of Plum Spreads

#### 2.7.1. Flow Measurement

Flow measurements were conducted using a Haake MARS rheometer (Thermo Scientific, Karlsruhe, Germany) with a Z20DIN measuring geometry at 20 °C. The sample was held at 20.00 °C for 300 s without applied shear to ensure thermal equilibrium before testing. In the next step, the shear rate was continuously increased from 0 to 100 s^−1^ over 120 s and then kept at 100 s^−1^ for an additional 120 s. Finally, the shear rate was decreased to 0 s^−1^ over 120 s. The degree of thixotropy, quantified by the area of the hysteresis loop (ΔA), was calculated from the resulting flow curves. Apparent viscosity (η, Pa·s) was measured from the flow curve and presented as a mean value at a shear rate of 100 s^−1^.

#### 2.7.2. Dynamic Oscillatory Measurements

Viscoelastic properties of examined plum spreads were determined using a Haake MARS rheometer (Thermo Scientific, Karlsruhe, Germany) equipped with serrated parallel plate geometry (PP35, 35 mm diameter, 1 mm gap). The sample was allowed to rest at 20 °C for 200 s with no applied stress (0.000 Pa) to ensure thermal equilibrium and remove residual stress before initiating the test. Oscillatory frequency sweep tests were conducted under controlled stress (CS) mode with a constant stress value of 1.000 Pa, which was within a linear viscoelastic region in the frequency range from 0.10 Hz to 10.00 Hz. This test is used for the determination of the material’s viscoelastic properties, such as storage modulus (G’) and loss modulus (G”), across a range of applied frequencies.

### 2.8. Textural Measurements

#### 2.8.1. Penetration Test

Textural analysis of the plum spreads was conducted using a TA.XTPlus Texture Analyzer (Stable Micro Systems, Godalming, UK) with a 1″ radius cylinder (P/1 R) probe to determine its mechanical properties [17]. Indicators of firmness were maximal force—MF (N) and work of penetration—WoP (N s), while the indicator of adhesiveness was WoA (N s). The total amount of force needed for the penetration process was WoP, and conversely, the total amount of force required for the withdrawal of the probe from the sample was WoA.

#### 2.8.2. Spreadability Test

Besides penetration tests, TA.XTPlus Texture Analyzer (Stable Micro Systems, Godalming, UK) was also used to determine plum spread spreadability. The spreadability was assessed using a 5 kg load cell and the TTC Spreadability RIG (HDP/SR), which consists of male and female Perspex conical elements. This textural analysis involves measuring the maximum force during compression of the upper element (male cone) through the sample placed in the lower element (female cone). The force value represents the firmness of the sample, F (N), while the total amount of force needed to shear the layers of the product during penetration signifies the shear work WoS (N s).

Before measurement, the height calibration was performed with a return distance of 25 mm. The instrument was run according to the following settings: pre-test speed at 1 mm/s, test speed at 3.0 mm/s, post-test speed at 10.0 mm/s, measurement distance of 23 mm, and a trigger force of 5 g.

### 2.9. Proximate Nutritional Composition of Semi-Industrial Plum Spreads

The protein and fat content were determined by the Kjeldahl method and the Soxhlet method, respectively [23]. The total sugar content was determined using the Luff-Schoorl method [24]. Ash content was analyzed according to the Association Official of Analytical Chemists (AOAC International, 2000) standard method 940.26 [25]. The total carbohydrates were determined as the difference: 100—(protein content + fats + fibers + moisture + ash) [26]. The Na content was analyzed using atomic absorption spectroscopy (AAS) following a dry-ashing process and was used to calculate the salt content in the product in accordance with EU Regulation No 1169/2011 [27]. The energy value of the plum products was calculated according to EU Regulation No 1169/2011 [27].

### 2.10. Statistical Analysis

The F-test was initially performed to assess the homogeneity of variances between two samples. Following this, a *t*-test was applied to evaluate the statistical significance of differences in the mean measurements between the two samples at a 5% significance level (*p* < 0.05). The Shapiro-Wilk test was used to check the normality of the data distribution among the tree spreads. When *p* > 0.05, ANOVA was performed; otherwise, the non-parametric Kruskal-Wallis test was applied. Post-hoc analysis for ANOVA included the Tukey HSD test (*p* < 0.05), and for the Kruskal-Wallis test, pairwise Mann-Whitney U tests were conducted (*p* < 0.05). STATISTICA 13.1 (TIBCO Software Inc., Palo Alto, CA, USA) was used to perform the analysis.

## 3. Results and Discussion

### 3.1. Chemical and Physical Characterization of Plum Materials

#### 3.1.1. Dietary Fibers and Pectin Substances of Plum Materials

Dietary fibers were determined in both raw materials, LPS and PP, with results presented in Table 2. The TDF content in plum purée was 15.14 g/100 g dry matter (d.m.) (1.90 g/100 g fresh weight, FW), which falls within the range of fiber concentrations typically found in plums (1.3–2.4 g/100 g FW) [28]. Dietary fibers accumulate primarily in the skin of the plum fruit [9], which is why TDF concentrations are much higher in lyophilized pomace derived from the exocarp of the plum. The TDF concentration in LPS was 38.98 g/100 g d.m.

The concentrations of IDF and SDF fractions varied between PP and LPS. The IDF and SDF values in PP were almost identical (Table 2), whereas in LPS, the IDF was the dominant fraction (24.81 g/100 g d.m.), compared to SDF (14.19 g/100 g d.m.). The soluble fraction is considerably less abundant in the skin than in the flesh of the plum fruit [8], and this trend was confirmed in the current study. The soluble fraction in PP (obtained from the whole fruit) makes up nearly 52% of the TDF content, which is consistent with previous findings for different plum varieties [8]. In LPS, the SDF/TDF ratio was 36%, which is higher than the 13–22% range reported by the same authors, depending on the plum variety.

Pectins are the dominant compounds in the soluble fiber fraction of dried plums [29], while cellulose is the predominant compound in the insoluble fiber fraction [30]. Determination of pectin fraction concentrations in the raw materials (Table 2) revealed that protopectin was the most abundant pectin substance in both PP and LPS. The concentration of protopectin in LPS was 3.08 g/100 g d.m., while in PP, the concentration was four times higher, 11.36 g/100 g d.m. (1.43 g/100 g FW). According to prior research, a lower amount of soluble pectin substances was found in the skin of the plum than in the flesh after enzyme-assisted juice extraction [8]. Although pectinase enzymes were not used in the production of pomace in this study, as reported in [8], the pectin content was lower in LPS compared to PP (Table 2). The pectin concentration in PP and LPS was 1.07 and 0.73 g/100 g d.m., respectively. The concentration of pectic acids in PP and LPS was found to be lower than that of pectin in the respective samples (Table 2).

#### 3.1.2. Water Retention Capacity and Structuring Potential of Lyophilized Plum Skin

The WRC of the LPS sample was assessed at room temperature, 82 °C (laboratory production conditions), and 50 °C (under vacuum) to evaluate its ability to bind water during cooking. The obtained results show that the WRC at room temperature (3.85 g/g) and at 82 °C (3.86 g/g) was nearly identical and slightly higher than at 50 °C (3.63 g/g), with no statistically significant difference (*p* > 0.05). Thus, the WRC of the LPS remained consistent across temperatures, with no significant variation (*p* > 0.05). The obtained values for LPS were higher compared to the WRC of black currant pomace (2.2 g/g) [31], dried raspberry and blueberry pomace (2.10 and 3.07 g/g) [32], and freeze-dried tomato pomace (3.10–3.58 g/g) [33], but lower than the WRC values for plum skin (5.5–7.1 g/g) and fruit (12.7–15.6 g/g) from three plum varieties studied [8]. The skin of the plum has sufficiently high WRC values, which makes it a suitable functional ingredient for use in the food sector [8]. Additionally, the presence of fibers in raw materials is essential for forming gel structures and enhancing the stable textural properties of products such as jams and yogurts by preventing syneresis [34]. Given that lyophilized pomace contains over 38% fiber and nearly 0.75% pectin in dry matter, LPS could be considered a potential gelling and structuring agent for functional plum spreads. It may also serve as an alternative to gelling agents in the development of low- or reduced-calorie spreads.

### 3.2. Nutritional Composition of Semi-Industrial Plum Spreads

The proximate nutritional composition of both the functional and control spread prepared in a vacuum cooker was determined and compared in order to investigate the impact of LPS integration on the spread’s structure, with a primary focus on dietary fiber and total sugar content. According to the nutrition and health claims outlined in EU Regulation (EC) 1924/2006) [35], the final F-IS qualifies to be labeled as “rich in fiber” (or “high in fiber”), as it contains a minimum of 6 g of fiber per 100 g (Table 3).

In addition, the functional product exhibited a sugar content reduction of over 30% compared to standard products, permitting it to meet the requirements of the “reduced sugar content” claim. The soluble dry matter, which is below 45%, corresponds to a jam with a reduced energy value [36]. Similarly, our products had 40 °Brix and 43 °Brix (F-IS and CS, respectively) [18], which allowed their classification as reduced-calorie products.

Furthermore, the functional product demonstrates a 35% reduction in energy value compared to commercial plum jams (≈1000 kJ/100 g), providing an advantage to the newly developed plum product in accordance with the aforementioned regulation. The energy value of the developed functional product is approximately 15% lower than that of the control product (Table 3).

Despite the promising nutritional claims and phenolic fortification [18], it is crucial to assess the acceptance of the functional spread. Future consumer testing will compare F-IS with commercial low-calorie and standard plum jams, focusing on how LPS affects the sensory profile. This will offer insights that could guide potential enhancements of the spread.

### 3.3. Texture Analysis

The textural properties of the developed products enriched with LPS (F-LS and F-IS) and the control spread (CS), which was prepared without the functional ingredient, are presented in Table 4. The behavior of all samples was tested during penetration with a probe, where the first three parameters were extracted from the “force-time” curve. Additionally, spreadability was determined for all samples to simulate the real process of consuming this type of food, such as spreading it on a slice of bread with a knife. The results obtained for MF, WoP, and WoA (absolute values) for the functional products are significantly higher (*p* < 0.05) than those of the control sample. In other words, the functional products exhibit higher firmness and adhesiveness, with the sample providing greater resistance to probe penetration.

The spreadability test shows the same trend as the indicators in the previous textural tests, as the F and WoS were significantly higher (*p* < 0.05) for both F-LS and F-IS compared to CS (Table 4). The increase in all investigated textural parameters was multiple times higher in the functional spreads than in the control. Furthermore, the semi-industrial vacuum-cooked spread demonstrated statistically significantly higher levels of all texture indicators compared to the one prepared in an open pan (F-IS and F-LS, respectively, Table 4). The compact packing of LPS particles into the microstructure of the functional spreads might be responsible for the strengthening of the gel-like fruit system [17,37]. Notably, the increase in dried plum pomace quantity in formulating an optimal spread in our previous study [17], from 2% to 10%, was accompanied by an increase in firmness and adhesiveness. Similar findings have been reported in studies using various fiber sources in the formulation of fruit jams, all leading to the same conclusion [2,38]. Culetu et al. pointed out that concentrating plum skin in the production of traditional sugar-free plum jams leads to increased system firmness [39].

The influence of LPS addition on textural properties might be a result of the hydrating properties of fibers or a consequence of the increase in fiber content in the spreads’ matrix, which strengthens the structure primarily built by pectin [2,38,40]. Despite the fact that the SDF/TDF ratio in LPS is lower than in PP, the higher concentration of the soluble fraction in the final product originates from LPS since LPS is used in its dried form.

Similar to SDF, pectin concentration also increased in samples where LPS was used, ensuring the necessary levels of this gelling agent to initiate the gel networking process. LPS was substituted with an equivalent amount of water in the CS, while the PP addition remained the same in the initial ingredient mixtures for all spreads (70%, Table 1). This means that more pectin was available in the functional products for gel-like structure formation compared to the control, where the only pectin source was PP. Theoretically, pectin from LPS concentrates to 0.81 g/100 g in the final product, whereas pectin from PP is anticipated to reach a concentration of 0.17 g/100 g (nearly 1%) in the functional spreads. On the other hand, in the control sample, only 0.23 g/100 g (theoretically) would be present in the matrix when cooked to the specified °Brix. Despite the possible degradation of pectic substances during the cooking of fruit, the water-soluble pectin remained unchanged during the thermal processing of apricots [41]. Assuming similar pectin behavior in our study, pectin from the LPS was sufficient to form a network in the structure of the functional product, as the minimum amount of pectin added as an additive for jams or similar products is around 1% [36]. Using the carbazole test, pectin concentrations of 0.39% and 0.83% were found in cherry and apricot jams, respectively [42]. In a recent study on plum jams prepared from plum skin purée, an increase in pectin concentration led to higher system consistency due to increased firmness [43]. Additionally, the increase in pectin concentration had the same effect on the viscosity index [43]. Protopectin has been reported as significant in preserving the firmness of heat-processed fruits [41]. Thus, protopectin from LPS might have a similar role in functional spreads to that of the current study.

### 3.4. Components and Functional Groups Detected in Lyophilized Plum Skin by ATR-FTIR

**Figure 1:**
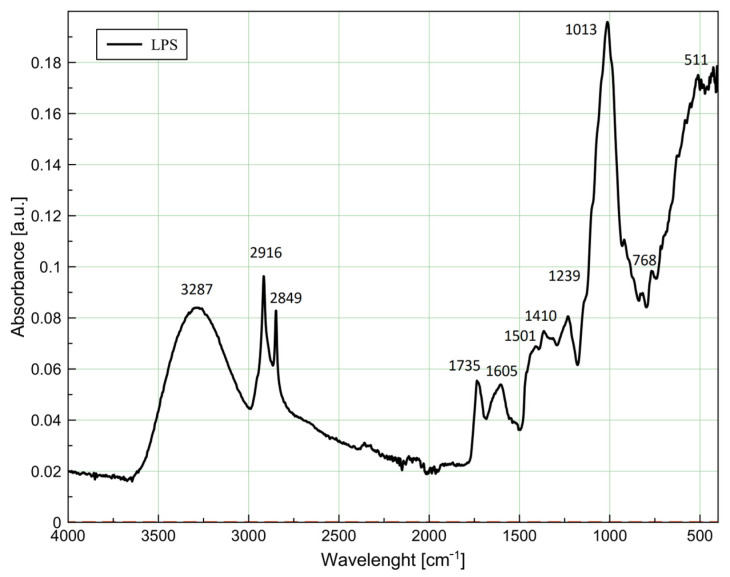
ATR-FTIR spectra of lyophilized plum skin (LPS).

The spectrum of the LPS sample (Figure 1) depicts three major regions, namely O–H stretching (centered at ~3300 cm^−1^), C–H stretching (3000–2800 cm^−1^) and the fingerprint region (<1500 cm^−1^), mostly related to carbohydrates.

The peak detected at 3287 cm^−1^ is associated with O–H stretching vibrations in polysaccharides and lignins, as was previously detected in grape seed extract [44]. The following medium peaks at 2916 and 2849 cm^−1^ were attributed to CH_2_ asymmetric stretching and symmetric stretching vibrations, respectively, in lignins and lipids [44,45].

The stretching vibrations of the carbonyl group associated with esterified pectin were detected at 1735 cm^−1^ [46], which is in line with the results on pectic substance content reported in Table 2. Similarly, the detected asymmetric stretching of COO- and C=C stretching at 1605 cm^−1^ were associated with present pectins and phenolic compounds [44] consistent with the pectic substance content detected herein as well as previously reported results on diverse phenolic compounds in plum skin [18]. In addition, peaks in the range 1500–1400 cm^−1^ were also associated with the presence of phenolic compounds, namely C=C stretching vibrations of the ring in the anthocyanins and flavones group [47]. The CH_2_ bending vibrations at 1365 cm^−1^ alongside C–O stretching and O–H bending vibrations at 1231 cm^−1^ were ascribed to polysaccharides, including dietary fibers and pectin [44] and further confirm the presence thereof in the sample (Table 2). The strongest absorption peak at 1013 cm^−1^ was attributed to C–O and C–C stretching in polysaccharides and pectins, as previously found for grape skin [44]. The following weak peaks at 818 and 768 cm^−1^ were assigned to the ring vibrations of anthocyanins [48], while the peak at 718 cm^−1^ was related to CH_2_ rocking vibrations in cutin and waxes [46]. Finally, peaks at 554 and 511 cm^−1^ were associated with C–C–C bending vibration in the A ring of the flavanone group [47].

### 3.5. Rheological Properties

**Figure 2:**
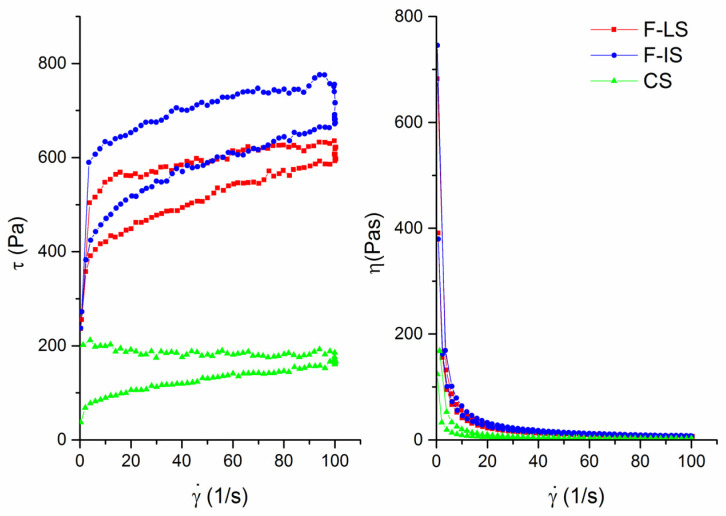
Flow curve measurements of F-LS—functional spread cooked in a laboratory environment, F-IS—functional spread cooked in a semi-industrial environment, CS—control spread cooked in a semi-industrial environment, τ (Pa)—shear stress, η (Pas)—viscosity, γ*˙* (1/s)—shear rate.

Rheological tests were performed to gain a deeper understanding of the influence of LPS on the spread’s structure. The results for the hysteresis loop area and apparent viscosity of all investigated spreads are shown in Figure 2 and Table 5. Similarly to textural properties, the obtained rheological parameters followed the same trend. Specifically, ΔA and η values were the highest in the F-IS spread, followed by the F-LS and CS spreads, with statistically significant differences observed among all samples (*p* < 0.05).

**Figure 3:**
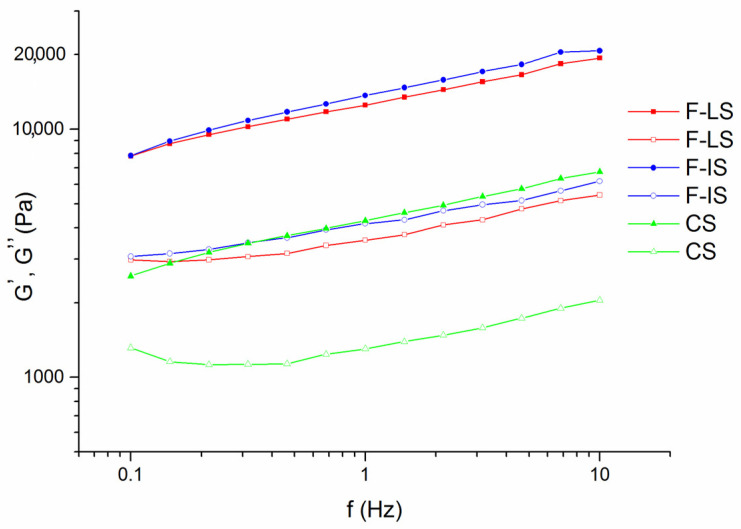
Mechanical spectra of F-LS—functional spread cooked in a laboratory environment, F-IS—functional spread cooked in a semi-industrial environment, CS—control spread cooked in a semi-industrial environment. Storage modulus (G’—full symbols), loss modulus (G”—empty symbols).

The storage modulus measures the reversible (elastic) structural changes, while the loss modulus reflects the irreversible (viscous) structural changes of the examined system [49]. All investigated samples have demonstrated typical viscoelastic gel-like behavior with the prevalence of elastic (G’) modulus over viscous (G”) modulus (Figure 3). The obtained moduli were frequency-dependent for all tested samples, which is a characteristic of weak gel-like systems [50].

Functional spreads exhibit differences in their textural and rheological properties in comparison to the control sample, with F-IS showing significantly higher values (*p* < 0.05) across all parameters compared to F-LS (Table 4 and Table 5). The obtained findings could be related to higher dietary fiber, as well as the pectin content of LPS-containing samples. According to Mohammadi-Moghaddam et al., the increase in pectin content resulted in the formation of a stiffer and stronger gel network due to the interaction between pectin with carbohydrates and crude fiber of plum peel [43]. Moreover, the results of water retention capacity revealed that LPS powder exhibited significant water-holding ability, leading to a reduced amount of free water in these systems compared to the control sample. Thus, water absorbed by fibers results in a viscosity increase and the prevention of syneresis [51]. The obtained results are in accordance with the findings of Figueroa and Genovese, who investigated the effect of different dietary fiber additions such as from apple, bamboo, psyllium and wheat, as well as pectin addition on physicochemical properties of products similar to fruit jams [52]. They concluded that at constant pectin concentration, the addition of fiber resulted in an increase in storage modulus in comparison to the control sample. The reason for such behavior was explained by the fact that the soluble dietary fiber fraction restricted the amount of free water by its gelling mechanism, whereas the insoluble fiber fraction acted as a filler in the gel matrix, resulting in the strengthening of the system structure.

The WRC was measured for LPS at both cooking temperatures (50 °C and 82 °C), but no significant differences were found (*p* > 0.05). The observed differences in characteristics between F-IS and F-LS may be attributed to the pasteurization step used in the production of F-IS, as both spreads were prepared using the same formulation. Pasteurization at 85 °C for 1 h may enhance the pectin level in F-IS due to the possible conversion of insoluble to soluble pectins. Thermal processing of pectinmethylesterase pretreated carrots leads to the conversion of insoluble pectin fraction into water-soluble soluble fraction [53]. Additionally, the cooking time for F-IS was longer than that for F-LS (45 min versus 10 min, respectively), which could have a similar effect on pectin content.

It should be noted that LPS is used solely in reduced-calorie products (F-IS) to meet consumers’ demand for low-calorie, low-fat, naturally sourced food ingredients [54]. The costs of producing LPS are mainly associated with the drying and milling processes. Expanding knowledge of the structure, chemical composition, and functionality of LPS pectin is possible through further extraction and purification of this material. Commercial pectins are mainly extracted from citrus peel and apple pomace, with high proportions of homogalacturonan regions (at least 65% GalA units; anhydrous D-galacturonic acid) required for technological applications [12]. The industrial extraction of pectin typically involves high temperatures and mineral acids, which can lead to degradation, loss of functionality, and decreased yields. Additionally, these methods contribute to environmental pollution and increase costs related to effluent treatment [12]. Non-conventional alternatives, such as subcritical water extraction, microwave, ultrasound, and enzyme-assisted extraction, or the use of “green” organic acids (citric, malic, and lactic acids), offer more sustainable and eco-friendly solutions [12,54]. However, their true economic benefits should be carefully evaluated to ensure they provide a cost-effective alternative to traditional extraction methods from fruit and vegetable by-products.

## 4. Conclusions

LPS proved to be a powerful structuring agent in functional plum spreads, enhancing both textural and rheological properties due to its high fiber and pectin content. LPS contains over 38% total dietary fiber and nearly 0.75% pectin in dry matter. The addition of LPS powder reflects its WRC and the gelling effect of pectin in functional spreads. Importantly, the reduced-calorie F-LS and F-IS were produced without the use of added pectin, relying solely on the natural source of pectin in LPS and PP (approximately 1% in total), which is sufficient to form a gelling network in semi-solid foods like fruit spreads. Thus, the use of LPS demonstrated the potential to fully replace structuring additives in commercial products, offering a natural alternative for reduced-calorie spreads and other foods where the technological functionality of fibers is required. The final plum spread contains more than 6% fiber, meeting the EU regulation for labeling as “rich in fiber”, thus contributing this unmatched macronutrient to consumers’ diets and health. Furthermore, the F-IS is green-labeled, as it contains no additives. Future research should focus on investigating the use of LPS in various food matrices (dairy, meat, bakery, etc.) as edible food coatings, as well as exploring its potential applications in pharmaceuticals (healing agents, supplements, cosmetics) as a structuring agent and source of dietary fibers.

## Figures and Tables

**Table 1:** Plum spread formulations and manufacturing conditions.

	Formulation (%, *w*/*w*)				Processing Conditions			
Spread	PP	Sucrose	LPS	Water	t (°C)	Pressure (bar)	Time (min)	Pasteurization
F-LS	70	15	10	5	82 ± 1	1.013	10	no
F-IS	70	15	10	5	50	−0.9	45	yes
CS	70	15	0	15	50	−0.9	90	yes

PP—plum purée, LPS—lyophilized plum skin, F-LS—functional spread cooked in a laboratory environment, F-IS—functional spread cooked in a semi-industrial environment, CS—control spread cooked in a semi-industrial environment.

**Table 2:** Chemical and physical characterization of plum materials.

Sample	Dietary Fibers (g/100 g d.m.)			Pectic Substances (g/100 g d.m.)		
	TDF	IDF	SDF	P	PA	PPC
PP	15.14 ± 0.32 ^a^	8.47 ± 0.12 ^a^	7.96 ± 0.17 ^a^	1.07 ± 0.01 ^a^	0.48 ± 0.02 ^a^	11.25 ± 0.21 ^a^
LPS	38.98 ± 0.52 ^b^	24.81 ± 1.16 ^b^	14.19 ± 0.66 ^b^	0.73 ± 0.03 ^b^	0.67 ± 0.03 ^b^	3.08 ± 0.08 ^b^

PP—plum purée, LPS—lyophilized plum skin, TDF—total dietary fiber, SDF—soluble dietary fiber, IDF—insoluble dietary fiber, P—pectin, PA—pectic acids, PPC—protopectin. Values designated by the same letter within a column are not significantly different (*p* > 0.05).

**Table 3:** Proximate nutritional composition of reduced calorie semi-industrial plum spreads (functional and control).

Nutrition Facts	F-IS	CS
	per 100 g:	per 100 g:
Energy (kJ/kcal)	655.78/155.14	759.75/179.13
Fat	0.185 g	0.155 g
-of which saturated	<0.1 g	<0.1 g
Carbohydrate	34.03 g	42.20 g
-of which sugars	33.15 g	41.11 g
Fiber	6.69 g	2.69 g
Protein	0.990 g	0.875 g
Salt	0.02 g	0.02 g

F-IS—semi-industrial functional plum spread with LPS; CS—semi-industrial control plum spread. Results are presented as mean values (*n* = 3).

**Table 4:** Textural properties of plum spreads.

	Penetration Test			Spreadability Test	
Spread	MF (N)	WoP (N s)	WoA (N s)	F (N)	WoS (N s)
**F-LS**	2.72 ± 0.10 ^a^	18.64 ± 0.65 ^a^	−2.93 ± 0.11 ^a^	11.52 ± 0.29 ^a^	11.81 ± 0.34 ^a^
**F-IS**	3.58 ± 0.26 ^b^	25.65 ± 1.13 ^b^	−4.12 ± 0.67 ^b^	14.64 ± 0.41 ^b^	15.67 ± 0.48 ^b^
**CS**	0.74 ± 0.03 ^c^	5.21 ± 0.37 ^c^	−1.09 ± 0.05 ^c^	3.42 ± 0.04 ^c^	2.97 ± 0.03 ^c^

F-LS—functional spread cooked in laboratory environment, F-IS—functional spread cooked in semi-industrial environment, CS—control spread cooked in semi-industrial environment, MF—Maximal Force (N), WoP—Work of Penetration (N s), WoA—Work of Adhesion (N s), F—Firmness (N), WoS—Work of Shear (N s). Values designated by the same letter within a column are not significantly different (*p* > 0.05).

**Table 5:** Parameters obtained from flow curve measurements.

Spread	Hysteresis Loop Area, ΔA (Pa/s)	η at 100 s^−1^ (Pa s)
F-LS	10,199.00 ± 740.28 ^a^	6.45 ± 0.18 ^a^
F-IS	14,636.67 ± 1090.89 ^b^	7.86 ± 0.47 ^b^
CS	6466.33 ± 341.79 ^c^	1.88 ± 0.11 ^c^

F-LS—functional spread cooked in a laboratory environment, F-IS—functional spread cooked in a semi-industrial environment, CS—control spread cooked in a semi-industrial environment, ΔA—hysteresis loop area, η—apparent viscosity. Values designated by the same letter within a column are not significantly different (*p* > 0.05).

## Data Availability

The original contributions presented in this study are included in the article. Further inquiries can be directed to the corresponding author.
